# Modest and Severe Maternal Iron Deficiency in Pregnancy are Associated with Fetal Anaemia and Organ-Specific Hypoxia in Rats

**DOI:** 10.1038/srep46573

**Published:** 2017-04-25

**Authors:** Andrew G. Woodman, Alison S. Care, Yael Mansour, Stephana J. Cherak, Sareh Panahi, Ferrante S. Gragasin, Stephane L. Bourque

**Affiliations:** 1 Department of Pharmacology, University of Alberta, Edmonton, Canada; 2 Department of Obstetrics and Gynaecology, University of Alberta, Edmonton, Canada; 3 Department of Anesthesiology & Pain Medicine, University of Alberta, Edmonton, Canada.

## Abstract

Prenatal iron-deficiency (ID) is known to alter fetal developmental trajectories, which predisposes the offspring to chronic disease in later life, although the underlying mechanisms remain unclear. Here, we sought to determine whether varying degrees of maternal anaemia could induce organ-specific patterns of hypoxia in the fetuses. Pregnant female Sprague Dawley rats were fed iron-restricted or iron-replete diets to induce a state of moderate (M-ID) or severe ID (S-ID) alongside respective controls. Ultrasound biomicroscopy was performed on gestational day (GD)20 to assess uterine and umbilical artery blood flow patterns. On GD21, tissues were collected and assessed for hypoxia using pimonidazole staining. Compared to controls, maternal haemoglobin (Hb) in M- and S-ID were reduced 17% (P < 0.01) and 48% (P < 0.001), corresponding to 39% (P < 0.001) and 65% (P < 0.001) decreases in fetal Hb. Prenatal ID caused asymmetric fetal growth restriction, which was most pronounced in S-ID. In both severities of ID, umbilical artery resistive index was increased (P < 0.01), while pulsatility index only increased in S-ID (P < 0.05). In both M-and S-ID, fetal kidneys and livers showed evidence of hypoxia (P < 0.01 vs. controls), whereas fetal brains and placentae remained normoxic. These findings indicate prenatal ID causes organ-specific fetal hypoxia, even in the absence of severe maternal anaemia.

Iron-deficiency (ID) is the most common nutritional deficiency worldwide^1^. ID anaemia, a condition in which circulating haemoglobin (Hb) levels are reduced, is estimated to affect 1.2 billion people globally^2^; the prevalence of latent ID, in which no overt signs of anaemia exist, is undoubtedly higher. One of the populations most at risk for ID and resultant anaemia is pregnant women, due blood volume expansion and demands from the fetal-placental unit^3^,^4^. Global rates of anaemia in pregnant women are estimated to be 38%^3^, with 22% of pregnant women in developed nations affected^5^, emphasizing the importance of iron status assessments in vulnerable groups regardless of geographical location.

ID during fetal and postnatal development is an important health concern that can have lasting effects on the offspring. ID during pregnancy and the postnatal developmental period causes altered growth trajectories, and is associated with long-term cognitive deficits^6^,^7^, cardiovascular perturbations^8^,^9^,^10^,^11^,^12^, and metabolic dysfunction^13^,^14^,^15^. Interestingly, iron supplementation and repletion of iron stores in children whose mothers had ID during pregnancy does not appear to alleviate the persistent health complications^16^, suggesting adequate iron supply to the fetus throughout gestation is critical. Despite its well-described programming effects, the mechanisms by which ID during pregnancy alters fetal growth trajectories are unknown.

Hb is the molecular vehicle responsible for carrying atmospheric oxygen from the lungs to the tissues. Under normal circumstances (i.e. a non-pregnant state), the amount of oxygen delivered to tissues exceeds demands by a factor of approximately 4, with the notable exception of the heart, which extracts approximately 50% of its delivered oxygen^17^. This physiological reserve, coupled with cardiovascular compensatory mechanisms that alter blood flow and increase cardiac output, maintain adequate tissue oxygenation even in cases of severe anaemia^18^. During pregnancy, oxygen demands in the fetus are undoubtedly high, corresponding to a lower physiological reserve of oxygen delivery to tissues. In the case of anaemia during pregnancy, it is not presently clear whether adaptive increases in cardiac output and changes in blood flow^19^ are capable of mitigating hypoxia caused by decreased arterial oxygen delivery to the fetus^20^,^21^. Although hypoxia inducible factor-1α (HIF-1α) protein has been shown to accumulate in placentae and hearts of anemic fetuses^22^,^23^,^24^, it is noteworthy that prolyl hydroxylase-activity is dependent on iron^25^, and as such increased HIF-1α expression may be caused by ID *per se,* rather than hypoxia. Despite this, no other studies have explored organ-specific patterns of hypoxia in the fetuses of ID mothers.

Together, these studies provide a rationale for investigating the relationship of prenatal ID and oxygen status within the fetus during gestation. In this study, we utilized two groups of rats at different ages to induce different degrees of maternal anaemia, to assess the relationships between maternal and fetal iron status and oxygen tension; these groups are designated moderate (M-ID) and severe (S-ID) iron deficient groups on the basis of maternal iron status at the end of gestation. Using these models, we sought to determine: (1) whether maternal and fetal blood flow patterns are altered by prenatal ID using ultrasound biomicroscopy; and (2) the organ specific patterns of hypoxia in dams and fetuses.

## Results

### Maternal and Fetal Outcomes

**Figure 1:**
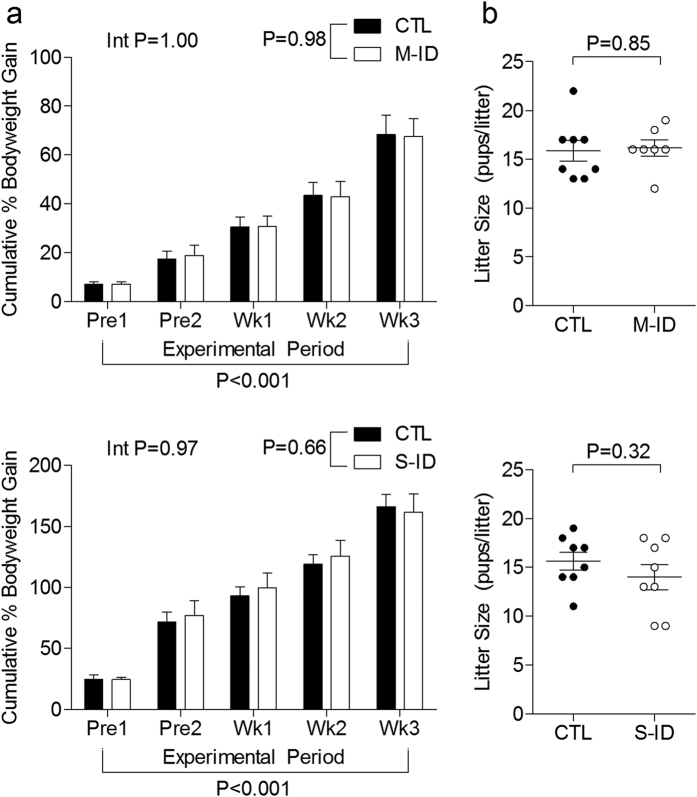
(**a**) Maternal cumulative weight gain over initial body weight and (**b**) litter sizes in moderate ID (M-ID) and severe ID (S-ID) groups. Top and bottom panels depict M-ID and S-ID data sets, respectively. Data is presented as mean ± SEM, and n = 7–8. In panel A, P values reflect 2-way ANOVA outcomes; in panel b, P values reflect Student’s *t*-test outcomes.

**Figure 2:**
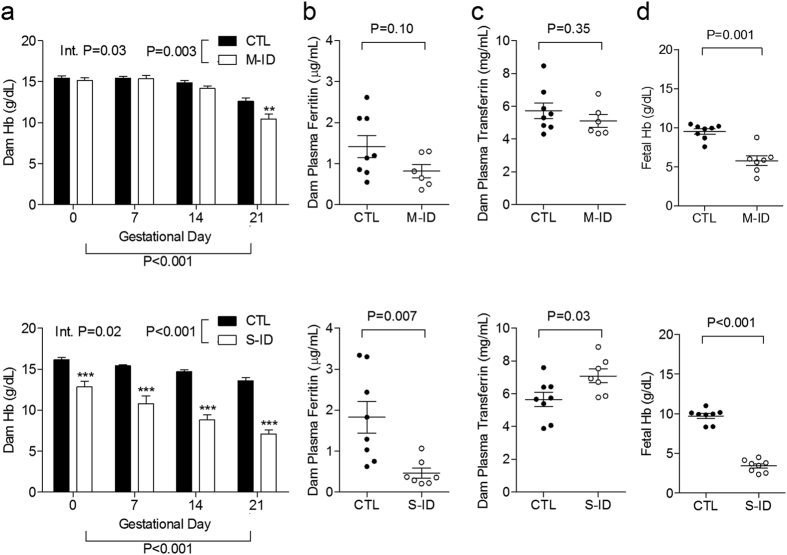
(**a**) Dam Hb, (**b**) dam plasma ferritin on GD21, (**c**) dam plasma transferrin on GD21, and (**d**) fetal Hb on GD21 in M-ID and S-ID groups. Top and bottom panels depict M-ID and S-ID data sets, respectively. Data is presented as mean ± SEM, and n = 6–8. In panel a, P values reflect 2-way ANOVA outcomes; in panels b–d, P values reflect Student’s *t*-test outcomes.

Maternal iron restriction had no impact on total food consumption in M-ID and S-ID groups (M-ID: 709.4 ± 13.3 g, controls: 698.1 ± 15.9 g, n = 5, P = 0.56; S-ID: 803.2 ± 29.2 g, controls: 840.4 ± 13.1 g, n = 8, P = 0.19), nor did it affect maternal cumulative weight gain or litter sizes (Fig. 1). Iron restriction in the M-ID group caused a 17% reduction in maternal Hb compared to controls by GD21 (Fig. 2a), with no apparent changes in maternal plasma ferritin (Fig. 2b) or transferrin (Fig. 2c). In contrast, maternal Hb in the S-ID group was 48% lower compared to controls (Fig. 2a), plasma ferritin levels were 75% lower than controls (Fig. 2b), and transferrin levels were 25% higher than controls (Fig. 2c). Fetuses in the M-ID group had Hb levels reduced by 39% compared to controls, whereas S-ID fetuses had Hb levels reduced by 65% (Fig. 2d).

**Figure 3:**
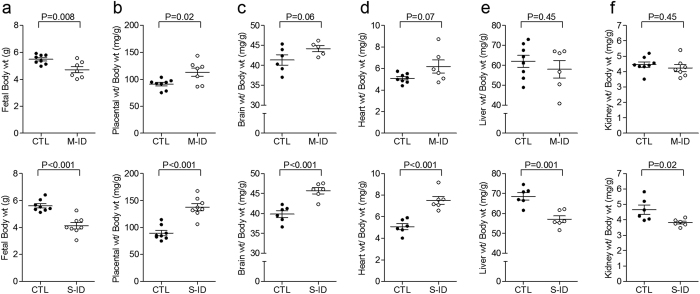
(**a**) Fetal bodyweights, (**b**) relative placental weights, (**c**) relative brain weights, (**d**) relative heart weights, (**e**) relative liver weights, and (**f**) relative kidney weights in M-ID and S-ID groups. Top and bottom panels depict M-ID and S-ID data sets, respectively. Data is presented as mean ± SEM, and n = 6–8. P values reflect Student’s *t*-test outcomes.

M-ID and S-ID resulted in 14% and 26% decreases in fetal bodyweight compared to controls, respectively (Fig. 3a). M-ID caused modest changes in fetal growth trajectories with increased relative placental size (Fig. 3b) and trends for increased brain (Fig. 3c) and heart weight (Fig. 3d), albeit no differences in liver or kidney weights were observed (Fig. 3e,f). The S-ID group was characterized by more pronounced alterations in fetal organs weight at the end of gestation, including increased relative placental (Fig. 3b), brain (Fig. 3c), and heart weight (Fig. 3d) which was accompanied by decreases in relative liver (Fig. 3e) and kidney weight (Fig. 3f).

### Maternal and Fetal Haemodynamics

**Figure 4:**
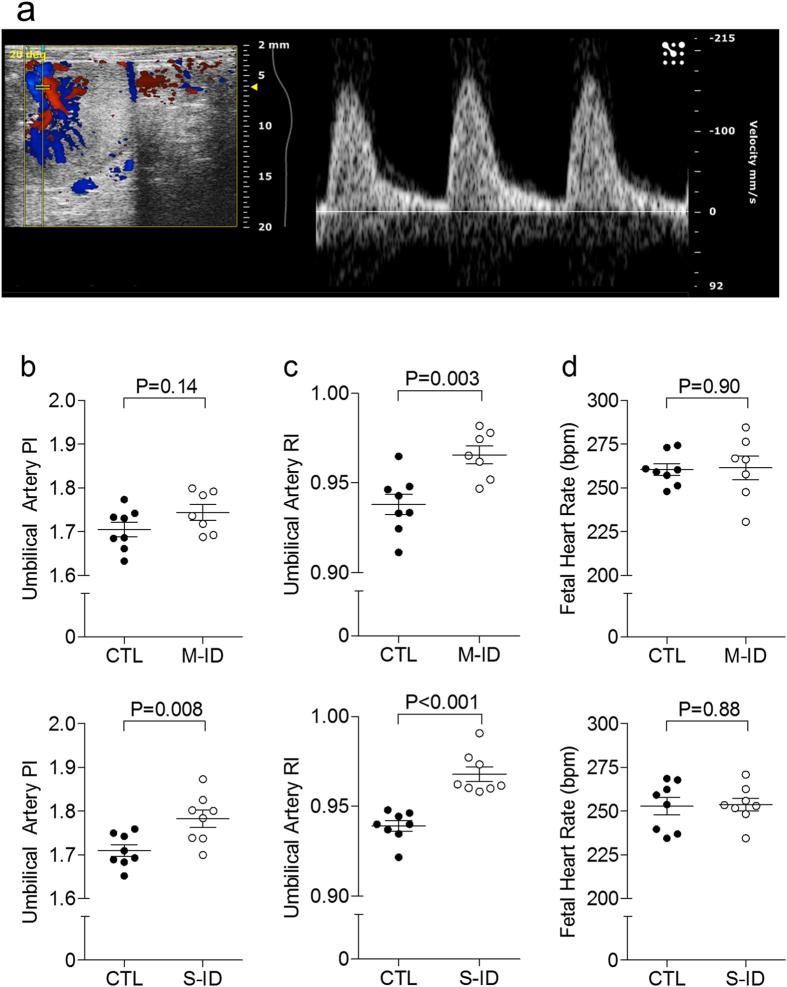
(**a**) Representative image of an umbilical artery Doppler from a moderate group control, (**b**) umbilical artery RI, (**c**) umbilical artery PI, and (**d**) fetal heart rate in M-ID and S-ID groups. Top and bottom panels in (**b**–**d**) depict M-ID and S-ID data sets, respectively. Data is presented as mean ± SEM, and n = 7–8. P values reflect Student’s *t*-test outcomes.

Neither M-ID nor S-ID dams exhibited alterations in uterine artery resistive index (0.60 ± 0.04 M-ID vs. 0.62 ± 0.03 controls, n = 7–8, P = 0.68; 0.62 ± 0.04 S-ID vs. 0.63 ± 0.02 controls, n = 8, P = 0.80) or pulsatility index (0.87 ± 0.08 M-ID vs. 0.91 ± 0.07 controls, n = 7–8, P = 0.69; 0.93 ± 0.04 S-ID vs. 0.91 ± 0.09 controls, n = 8, P = 0.88). However, fetal umbilical artery RI was increased in both groups (Fig. 4b) compared to respective controls, with no change in fetal heart rates (Fig. 4d). Umbilical artery PI was not changed in M-ID dams, whereas it increased in S-ID dams with respect to controls (Fig. 4c). Underlying these changes, umbilical artery PSV was elevated by more than 35% in both the M- and S-ID groups with respect to controls (270.3 ± 26.7 mm/s M-ID vs. 164.1 ± 12.3 mm/s controls, n = 7–8, P = 0.002; 287.0 ± 13.2 mm/s S-ID vs 216.4 ± 15.3 mm/s controls, n = 8, P = 0.004). The S-ID group also has a trend for decreased EDV versus controls (9.9 ± 1.4 mm/s S-ID vs. 13.1 ± 1.0 mm/s controls, n = 8, P = 0.08), whereas the M-ID group did not (10.3 ± 2.0 mm/s M-ID vs. 10.5 ± 1.0 mm/s controls, n = 7–8, P = 0.96). Core body temperature, measured during ultrasound assessments, was not different between groups (35.1 ± 0.3 °C M-ID vs. 35.3 ± 0.3 °C controls, n = 7–8, P = 0.72; 35.0 ± 0.3 °C S-ID vs 34.7 ± 0.3 °C controls n = 7–8, P = 0.44).

### Hypoxyprobe Staining

**Figure 5:**
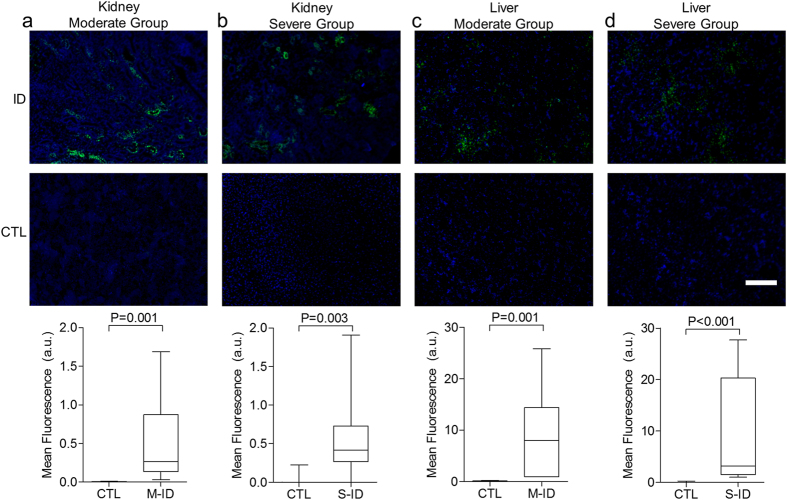
Hypoxyprobe staining in fetal (**a**) moderate group kidney, (**b**) severe group kidney, (**c**) moderate group liver, and (d) severe group liver. Top and bottom panels depict representative staining and quantification, respectively. The white scale bar represents 75 μm of tissue. Data are presented as median, quartiles, and range. P values reflect Mann-Whitney *U* test outcomes, n = 7–8.

**Figure 6:**
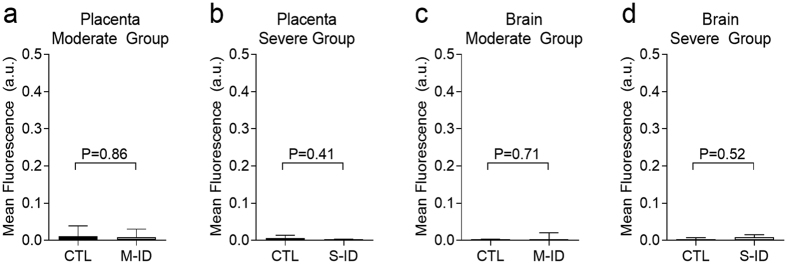
Hypoxypobe staining in fetal (**a**) moderate group placentae, (**b**) severe group placentae, (**c**) moderate group brain, and (**d**) severe group brain. Data are presented as median, quartiles, and range. P values reflect Mann-Whitney *U* test outcomes, n = 7–8.

**Figure 7:**
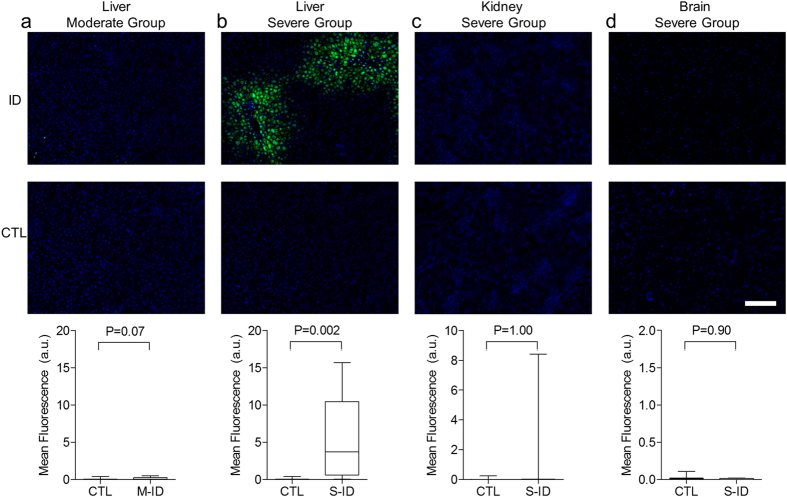
Hypoxyprobe staining in maternal (**a**) M-ID liver, (**b**) S-ID liver, (**c**) S-ID kidney, and (**d**) S-ID brain. Top and bottom panels depict representative staining and quantification, respectively. The white scale bar represents 75 μm of tissue. Data are presented as median, quartiles, and range. P values reflect Mann-Whitney *U* test outcomes, n = 7–8.

Evidence of fetal hypoxia, as assessed by pimonidazole staining, was similar in both M-ID and S-ID groups. Pimonidazole staining was more pronounced in kidneys and livers of both M-ID and S-ID fetuses, relative to their respective controls (Fig. 5). No pimonidazole staining was observed in fetal brain or in placentae of either group (Fig. 6). Finally, increased levels of staining were observed in dam liver tissues in the S-ID group, which was not evident in the M-ID group (Fig. 7a,b). No staining was observed in S-ID dam kidneys (Fig. 7c) or brains (Fig. 7d), and therefore these analyses were not performed in M-ID dams.

## Discussion

In this study, we investigated the effects of maternal iron restriction during pregnancy to assess the impact on growth, iron status, and hypoxia in the developing fetus. To summarize, we report that prenatal ID caused: (1) asymmetric fetal growth restriction, which was dependent on the severity of fetal anaemia; (2) no changes in uterine artery blood flow patterns, but altered umbilical artery blood flow patterns; (3) hypoxia in fetal livers and kidneys, but not in the brain or placenta. Taken together these findings suggest that ID, which may or may not manifest as overt maternal anaemia by the end of gestation, causes fetal anaemia and tissue-specific patterns of hypoxia, which may have important implications in the programming of long-term health in the offspring.

The M-ID rat model used in this study is one in which ID develops in the dam over the course of gestation, and only begins to show signs of anaemia at the end of pregnancy. In contrast, reductions in maternal Hb are observed throughout gestation in the S-ID group, which caused more pronounced fetal growth asymmetry. The M-ID group is intended to mimic a common clinical scenario in which the increased demands of pregnancy coupled with insufficient iron intake or poor gastrointestinal absorption^26^,^27^ causes the gradual depletion of iron stores, which can proceed without clinical manifestations until the end of gestation. As blood volume expansion continues and iron demands from the fetal-placental unit increase throughout gestation, reduced maternal Hb levels become apparent. The M-ID group models this clinical scenario well, as maternal Hb levels reside within the range considered normal in non-pregnant women (12–16 g/dL^28^), and only reach a modest degree of anaemia in the last week of gestation (<11 g/dL^29^). In the M-ID dams, we observed no changes in plasma ferritin or transferrin levels, which is in contrast to the S-ID model, further demonstrating that this treatment regimen produces a modest insult more likely to be overlooked in the clinical setting. Whereas the S-ID fetuses exhibited severe growth restriction and marked changes in organ grown patterns, the M-ID fetuses group exhibited lesser growth restriction, and no marked alterations in certain organ weights. However, we did observe subtle alterations in brain and heart weights (relative to body weight; Fig. 3), which likely reflects cardiovascular adaptations contributing to a brain-sparing effect (see below), suggesting this moderate anaemia does in fact cause iron depletion in the developing fetus by the end of pregnancy. Moreover, Mihaila *et al*. reported differences in embryo iron content as early as embryonic day 15 using a rat model of iron deficiency of similar severity as the M-ID group used herein^30^, suggesting that the fetus is exposed to a prolonged period of stress in the absence of maternal anaemia.

As noted above, ID *per se* can upregulate endogenous markers generally associated with hypoxia, such as HIF-1α^25^,^31^ and can therefore confound hypoxia assessments. The present study therefore relies on the suitability of exogenous pimonidazole as a more accurate marker of hypoxia. In non-ID contexts, pimonidazole staining correlates well with HIF-1α and other endogenous markers of hypoxia^32^,^33^, suggesting it is well-suited to detect physiologically relevant degrees of hypoxia (∼10 mmHg)^33^,^34^.

The majority of oxygen transported in the blood is bound to Hb, and consequently anaemia has a direct and profound impact on oxygen carrying capacity^20^,^35^. S-ID dams, unlike M-ID dams, exhibited signs of liver hypoxia at the end of pregnancy, indicating compromised oxygen transport and an inability to fully compensate via increases in blood flow. In the case of S-ID fetuses, concomitant reductions in both maternal and fetal Hb predictably resulted in hypoxia in certain tissues (e.g. kidneys, liver) but not in others (i.e. brain). Although blood gas parameters were not assessed in the present study owing to technical limitations, Darby *et al*. previously showed in an ovine model that a similar degree of fetal anaemia as seen in the present study causes reduced fetal blood O_2_ content, with changes in fetal blood pH and pCO_2_ 21. These hypoxia patterns are consistent with altered organ growth patterns, which may be indicative of fetal cardiovascular compensatory mechanisms. Indeed, coronary and cerebral blood flow in ovine fetuses tend to increase disproportionally in chronic anaemia^36^ suggesting a redistribution of blood flow towards the brain and heart at the expense of other organs (e.g. liver and kidney). Bastian *et al*. also reported increased blood vessel branching and elevated expression of angiogenic and vasculogenic genes in various brain regions^37^, suggesting other compensatory mechanisms are involved in the mitigating the hypoxia induced by anaemia.

Interestingly, in contrast with previous reports in ovine models of fetal anaemia^19^,^35^, no compensatory increases in fetal heart rate were observed in either the M or S-ID group. Although this could be attributed to the use of isoflurane anesthesia (the studies by Mostello *et al*. and Davis *et al*. were done in non-anesthetized sheep), it is noteworthy that anaemia-induced increases in cardiac output are thought to stem largely from decreases in blood viscosity (resulting primarily in reduced afterload) and increased contractility^19^ rather than autonomic nervous system-driven changes in cardiac function^38^. In this regard, it is not surprising that fetal heart rates were not affected. However, whether fetal cardiac responses to anaemia differ from adults, and whether there are developmental differences between rats and sheep in heart rate regulation^35^,^39^ may provide some insights into the mechanisms of fetal cardiovascular compensation to anaemia.

The marked increase in placental size in both the M-ID and S-ID groups suggest that the placenta is sensitive to oxygen (or iron) deficiencies, despite no increases in blood flow with anaemia^19^,^36^. Although the consequences of this excessive placental growth are not clear, this may reflect a compensatory mechanism to increase oxygen and nutrient exchange and therefore offset the reduced oxygen delivery caused by lower maternal and fetal Hb levels and mitigate a hypoxic insult to the fetus. However, Lewis *et al*. reported that prenatal ID is associated with reduced vascularity and capillary volume in the placenta^40^, and the authors speculate that the alterations in placental morphology (and possibly abnormal vascular structure/function) may be a cause of altered fetal growth trajectories in this model. As such, increased umbilical artery resistive index in both M-ID and S-ID groups may reflect an abnormal placental vascular development, and may be a cause of altered fetal growth trajectories, rather than a beneficial adaptive mechanism. Moreover, our results showing that S-ID, but not M-ID, contributes to an elevated PI may indicate of a greater level of pathology with increasing severity of ID.

Our study implicates fetal hypoxia as a possible mechanism that contributes to the programming of chronic diseases in later life. We have previously shown that perinatal ID can induce long-term health complications, ranging from cardiovascular perturbations^12^, to increased propensity for obesity and metabolic dysfunction^14^,^15^, to cognitive deficits^7^. These effects are said to be ‘programmed’ because the phenotypic and functional consequences persist long after the stressor is removed. The observation that fetal kidneys become hypoxic may be a contributing factor to the cardiovascular dysfunction seen in adult PID offspring, which is characterized by reduced nephron endowment^41^, hypertension^8^,^9^,^11^, and alterations in the intrarenal haemodynamics associated with salt sensitivity^12^. Consistent with this hypothesis, prenatal hypoxia has also been shown to cause reduced nephron endowment in rats^42^, and result in hypertension in adult offspring^43^.

Although prenatal hypoxia has been shown to induce similar programming effects on cardiovascular and metabolic function as prenatal ID, the precise role of hypoxia *per se* in our M-ID and S-ID models requires further investigation. Persistent neurological deficits, including long-term changes in neurotransmitter function, cognition and behaviour^44^,^45^ are well described in models of perinatal ID anaemia, yet no evidence of brain hypoxia was observed in the present study. Thus alternative mechanisms (e.g. changes in functional iron levels, alterations in inflammatory and oxidant status, perturbations in energy metabolism^22^,^46^,^47^,^48^) may also be implicated in the pathophysiology of prenatal ID. Given the intimate relationships between iron, oxygen transport, energy, and inflammation, it is likely that the multiple mechanisms are involved. Moreover, we cannot discount the possibility that the compensatory mechanisms that mitigate the development of hypoxia are implicated in the programming of altered nervous system function. For example, hypoxia-induced increases in cerebral blood flow can result in blood brain barrier leakiness that accompanies accelerated vasculogenesis/angiogenesis^49^. In prenatal ID fetal rats, vasculogenic and angiogenic mediators implicated in this process are shown to be upregulated^37^, potentially implicating compromised integrity of the fetal blood brain barrier– a condition associated with a numerous of brain pathophysiologies^50^.

In summary, we have demonstrated that ID results in an organ-specific pattern of fetal hypoxia, and this can even occur in the absence of marked changes in maternal iron status. While it is well-established that prenatal ID is linked to long-term health outcomes, our results suggest the mechanisms underlying the programming of cardiovascular, metabolic, and neurological function in the offspring may be different based on the heterogeneous patterns of hypoxia within the fetus. Indeed, longstanding assumptions regarding the patterns of organ injury/dysfunction in the context of prenatal ID may be incorrect in the case of the fetal brain, in which no evidence of hypoxia observed, but may to be warranted in the case of other organs (e.g. liver and kidney). Although we recognize that there are inherent limitations to the applicability of these results to human pregnancies, our findings nevertheless emphasize the potential pitfalls of relying exclusively on maternal iron status as a predictor of fetal pathology, given that maternal anaemia can occur at the end of gestation, or may not appear at all. Consequently, alternative diagnostic markers of fetal ID and anaemia are needed so interventions can be instituted before these fetal complications alter developmental trajectories and cause long-term health consequences in the offspring.

## Methods

### Animals and Treatments

The experimental protocols described herein were approved by the University of Alberta Animal Care Committee in accordance with the guidelines established by the Canadian Council for Animal Care. Thirty-two female Sprague Dawley rats (16 at 12 weeks old, and 16 at 6 weeks old) were purchased from Charles River (Saint-Constant, Quebec, Canada) and housed in the University of Alberta Animal Care Facility. Dams had *ad libitum* access to food and water throughout the study. The animal care facility maintained a 12-hour light/dark cycle and an ambient temperature of 23 °C.

Two weeks prior to mating, dams from both age groups were randomly assigned to receive either a control or an iron-restricted diet; the two diets, based on the AIN-93G formula (Research Diets Inc.)^51^, were identical in composition with the exception that control diets (D10012G) contained 35 mg/kg iron (in the form of ferric citrate), whereas the ID diet (D15092501) had no added ferric citrate and contained only trace amounts of iron (3 mg/kg). This approach allowed us to feed dams an identical diet, but generate different degrees of ID anaemia in the dams, such that feeding the 6 week old female rats fed an iron-restricted diet generates a phenotype far more severe than that which occurs in 12 week old female rats fed the same iron-restricted diet. After two weeks on their respective diets, females were naturally bred (i.e. without synchronization of estrus) to age-matched males fed a standard iron-replete rodent chow (PicoLab 5L0D) by housing 1 male with 2 dams each night. Pregnancy was confirmed by the presence of sperm in a vaginal smear the following morning; this was considered gestational day(GD)0. Dams were housed individually starting on GD0, and food consumption, body weight, and Hb were assessed weekly. Maternal Hb levels was assessed using a HemoCue 201+ system from blood (∼10 μL) collected via saphenous venipuncture.

### Ultrasound Biomicroscopy

On GD20, ultrasound biomicroscopy was performed to assess uterine artery and fetal umbilical artery haemodynamic parameters, as previously described^52^. Rats were anaesthetized with isoflurane (5% induction, 2.5% maintenance in 100% O_2_). The abdomen was shaved and pre-warmed gel was used as an ultrasound coupling medium. Rats were imaged transcutaneously using an ultrasound biomicroscope (model Vevo 2100, VisualSonics, Toronto, ON, Canada) with a 16–21 MHz MicroScan array transducer probe. A 0.2 to 0.5 mm pulsed Doppler gate was used, and the angle between the Doppler beam and the vessel was <45°. Doppler waveforms were obtained from uterine arteries near the lateral-inferior margin of the utero-cervical junction close to the iliac artery on each side, as well as from the umbilical artery from three fetuses per dam. Peak systolic velocity (PSV) and end diastolic velocity (EDV) averages were obtained from a minimum of three consecutive cardiac cycles. Both resistive index (RI = [PSV − EDV]/PSV) and pulsatility index (PI = [PSV − EDV]/time averaged velocity [TAV]) were calculated.

### Tissue Collection and Analysis

After a minimum of 4 hours of recovery from anesthesia from ultrasound biomicroscopy assessments, rats were administered pimonidazole (60 mg/kg) by oral gavage. The next morning (GD21; term = GD22) dams were anesthetized (isoflurane, 5% induction, 3% maintenance in 100% O_2_) and blood was collected from the inferior vena cava into EDTA coated tubes, and centrifuged at 1500× g to isolate plasma. Rats were then euthanized by exsanguination and subsequent excision of the heart. Fetuses and their placentae were quickly removed, cleaned and weighed. Fetuses were decapitated and blood was collected for blood Hb assessments (HemoCue 201 + system). Fetal tissues were excised into ice-cold saline, cleaned, blotted dry and weighed; tissues were then fixed in 4% neutral-buffered formalin for 24 hours and subsequently embedded for histology as described^53^. Plasma transferrin and ferritin were assessed using kits (ab137993 and ab157732, Abcam), in duplicate for each dam, according to the manufacturer’s instructions.

For hypoxyprobe analysis, embedded tissues from dams and male fetal offspring were sectioned at 6 μm. Maternal brain and kidneys were sampled from the cortical regions, whereas the entire cross-sections of fetal tissues were used (due to their small size). Following rehydration and antigen retrieval in sodium citrate buffer (10 mM sodium citrate, 0.05% Tween-20, pH 6.0) for 20 min at 90 °C, tissues were blocked using 1% bovine serum albumin in tris-buffered saline (50 mM Tris, 150 mM NaCl, 0.1% tween 20, pH 9.0). Sections were then probed with FITC-preconjugated hypoxyprobe mouse monoclonal antibodies (HP61000 Kit, Hypoxyprobe Inc.) in blocking buffer at a dilution of 1:50 (0.01 mg/mL) and incubated overnight at 4 °C. After washing in tris-buffered saline, sections were mounted in DAPI-containing medium (Vectashield H-1200). Fluorescence intensities were measured using an Olympus IX81 fluorescent microscope. For each sample analyzed, mean fluorescence intensity was calculated from 6 separate fields of view. Thresholds of fluorescence images were normalized to average intensity of control group fluorescence (low threshold) and maximal fluorescence intensity of all samples (high threshold).

### Statistical Analyses

In all cases, the n values reflect the number of litters (or dams) used, and not the number of fetuses. When multiple pups were sampled from the same litter, mean values were calculated and considered n = 1; this corresponds to 1 pup per n in hypoxyprobe data sets, and 2–6 pups for all other data sets presented. Parametric data (i.e. food intake, body weight gain, organ weights, and haemodynamic variables) were analyzed by unpaired Student’s *t* test, or two-way analysis of variance with Bonferroni *post-hoc* tests; these data are presented as scatter plots with mean ± SEM. Non-parametric data (i.e. pimonidazole staining) were analyzed by Mann-Whitney *U*-test; these data are presented as bar and whisker plots showing as median, 25% and 75% quartiles, and range. Hypoxyprobe analyses were conducted in male fetal tissues, however trends were confirmed in select female tissues. All statistical analyses were conducted using Prism 5.0 (GraphPad Software, Inc.). P < 0.05 was considered statistically significant.

## Additional Information

**How to cite this article**: Woodman, A. G. *et al*. Modest and Severe Maternal Iron Deficiency in Pregnancy are Associated with Fetal Anaemia and Organ-Specific Hypoxia in Rats. *Sci. Rep.* **7**, 46573; doi: 10.1038/srep46573 (2017).

**Publisher's note:** Springer Nature remains neutral with regard to jurisdictional claims in published maps and institutional affiliations.
